# Assistência pré-natal: uma análise temporal utilizando as informações da *Pesquisa Nacional de Saúde* de 2013 e 2019

**DOI:** 10.1590/0102-311XPT143424

**Published:** 2025-06-27

**Authors:** Narayani Martins Rocha, Wanessa da Silva de Almeida, Paulo Roberto Borges de Souza, Célia Landmann Szwarcwald

**Affiliations:** 1 Instituto de Comunicação e Informação Científica e Tecnológica em Saúde, Fundação Oswaldo Cruz, Rio de Janeiro, Brasil.

**Keywords:** Cuidado Pré-Natal, Saúde Materno-Infantil, Serviços de Saúde Materno-Infantil, Pesquisa sobre Serviços de Saúde, Inquéritos Epidemiológicos, Prenatal Care, Maternal and Child Health, Maternal-Child Health Services, Health Services Research, Health Surveys, Atención Prenatal, Salud Materno-Infantil, Servicios de Salud Materno-Infantil, Investigación sobre Servicios de Salud, Encuestas Epidemiológicas

## Abstract

A assistência ao pré-natal prestada de forma inadequada está relacionada à ocorrência de desfechos negativos para mães e recém-nascidos, sendo fundamental seguir as diretrizes do Ministério da Saúde para garantir a qualidade do cuidado. O objetivo deste estudo foi analisar indicadores relacionados ao pré-natal utilizando as informações das edições da Pesquisa Nacional de Saúde (PNS) de 2013 e 2019. A população-alvo do estudo incluiu mulheres que tiveram filhos nos dois anos anteriores às pesquisas. Foram elaborados indicadores relacionados ao atendimento pré-natal, tais como número de consultas, exames realizados, monitoramento clínico-obstétrico e orientações recebidas. As prevalências dos indicadores e seus respectivos intervalos de 95% de confiança foram estimados e comparados entre 2013 e 2019, por meio do teste de qui-quadrado de Pearson, ajustado pela correção de Rao-Scott - que leva em consideração o desenho amostral. Houve aumento significativo no número de consultas de pré-natal realizadas (p < 0,05), bem como na realização de exames clínico-obstétricos (p < 0,05). Em relação à proporção de mulheres que receberam orientações sobre o serviço de referência para o parto, houve aumento significativo de 75% para 83% (p < 0,001), assim como a realização de testes para sífilis, de 66% para 79% (p < 0,001), e HIV, de 95% para 99% (p < 0,001). A análise da assistência ao pré-natal revela avanços importantes na cobertura e na qualidade do atendimento às gestantes no Brasil. No entanto, desafios persistem, como o início tardio do pré-natal e a insuficiente orientação sobre os serviços de referência para o parto.

## Abstract (en)

Inadequate prenatal care is associated with adverse outcomes for both mothers and newborns, making adherence to the Brazilian Ministry of Health’s guidelines essential for ensuring quality care. This study aimed to analyze indicators related to prenatal care using data from the 2013 and 2019 editions of the *Brazilian National Health Survey*. The target population comprised women who had given birth in the two years preceding each survey. Indicators related to prenatal care were developed, including the number of visits, performance of exams, clinical-obstetric monitoring, and the guidance provided. The prevalences of these indicators and their corresponding 95% confidence intervals were estimated and compared between 2013 and 2019 using the Pearson chi-squared test, adjusted by the Rao-Scott correction to account for the sampling design. A significant increase was observed in the number of prenatal visits (p < 0.05) and in the performance of clinical-obstetric exams (p < 0.05). Moreover, the proportion of women who received guidance on the referral service for delivery increased significantly from 75% to 83% (p < 0.001), as did the testing rates for syphilis (from 66% to 79%, p < 0.001) and HIV (from 95% to 99%, p < 0.001). The analysis reveals important progress in both the coverage and quality of prenatal care in Brazil. However, challenges remain, including the late initiation of prenatal care and insufficient guidance regarding referral services for delivery.

## Resumen

La atención prenatal inadecuada está relacionada con la ocurrencia de resultados negativos para las madres y los recién nacidos, y es esencial seguir las orientaciones del Ministerio de Salud para garantizar la calidad de la atención. El objetivo de este estudio fue analizar indicadores relacionados con la atención prenatal a partir de datos de las dos ediciones de la *Encuesta Nacional de Salud*, 2013 y 2019. La población objetivo del estudio incluyó a mujeres que tuvieron hijos en los dos años anteriores a las encuestas. Se desarrollaron indicadores relacionados con la atención prenatal, como el número de consultas, exámenes realizados, seguimiento clínico-obstétrico y orientación recibida. La prevalencia de los indicadores y sus respectivos intervalos de confianza del 95% se estimaron y se compararon entre 2013 y 2019, mediante el uso de la prueba de chi-cuadrado de Pearson, ajustada por la corrección de Rao-Scott, que tiene en cuenta el diseño de la muestra. Hubo un aumento significativo en el número de consultas prenatales realizadas (p < 0,05) y en la realización de exámenes clínico-obstétricos (p < 0,05). La proporción de mujeres que recibieron orientación sobre el servicio de referencia para el parto tuvo un aumento significativo del 75% al 83% (p < 0,001), así como sobre la realización de pruebas para sífilis, del 66% al 79% (p < 0,001), y de VIH, del 95% al 99% (p < 0,001). El análisis de la atención prenatal revela importantes avances en la cobertura y calidad de la atención a las mujeres embarazadas en Brasil. Sin embargo, todavía existen desafíos, como el inicio tardío de la atención prenatal y la orientación insuficiente sobre los servicios de derivación para el parto.

## Introdução

O pré-natal, instituído pelo Programa Nacional de Humanização do Parto e Nascimento (PHPN), estabelece diretrizes para o acompanhamento das gestantes, tais como: início no primeiro trimestre; realização de seis ou mais consultas; exames laboratoriais e procedimentos clínico-obstétricos; atividades educativas de promoção, imunização, atendimento multiprofissional e orientações sobre aleitamento materno; e parto ^1^ ^,^ ^2^ ^,^ ^3^ ^,^ ^4^ ^,^ ^5^. O cumprimento integral dessas diretrizes requer elementos facilitadores e acessíveis para garantir a adequação da assistência e o efeito protetor do pré-natal. O acompanhamento da gestante desde o primeiro trimestre é essencial para prevenir e identificar riscos que possam afetar a saúde materna ou fetal, assegurando a qualidade da assistência ^6^ ^,^ ^7^ ^,^ ^8^ ^,^ ^9^. A realização de todos os procedimentos preconizados contribui para reduzir a morbimortalidade materno-infantil, promover melhor crescimento intrauterino, um peso adequado ao nascer e menor ocorrência de prematuridade e, no que se refere às mães, menor frequência de complicações durante a gestação e no momento do parto ^9^ ^,^ ^10^.

A assistência ao pré-natal prestada de forma inadequada está relacionada à ocorrência de desfechos negativos para mães e recém-nascidos ^3^ ^,^ ^11^. Fatores como presença de intercorrências durante a gestação (eclâmpsia, diabetes, entre outros) associam-se a complicações maternas ^12^, e fatores relacionados ao recém-nascido são fatores de riscos fortemente associados à mortalidade neonatal (0-28 dias) no Brasil ^13^ ^,^ ^14^ ^,^ ^15^ ^,^ ^16^. Em sua maioria, esses fatores são considerados como evitáveis e refletem a assistência de saúde prestada à gestante, nos períodos pré-parto e parto, e ao recém-nascido ^14^.

A taxa de mortalidade infantil (TMI) é um importante indicador das condições de vida da população, como também da qualidade da atenção pré-natal, ao parto e ao recém-nascido ^17^. A redução da TMI faz parte dos Objetivos de Desenvolvimento Sustentável (ODS), do qual o Brasil é signatário e tem como uma das metas acabar com as mortes evitáveis de recém-nascidos e crianças menores de cinco anos ^18^.

Segundo dados do Sistema de Informações sobre Mortalidade (SIM) de 2021, cerca de 70% das mortes infantis ocorreram no período neonatal ^19^. Estudo realizado em 2015 mostrou que as afecções perinatais representavam o principal grupo de causas de morte, correspondendo a mais da metade dos óbitos infantis ^20^. Assim, o padrão de comportamento da mortalidade infantil no Brasil evidencia a importância dos fatores ligados à gestação, ao parto e ao pós-parto - preveníveis por meio de assistência à saúde de qualidade. Apesar das altas coberturas de pré-natal e parto hospitalar, é essencial promover maior integração entre as ações desenvolvidas na atenção primária e os serviços de atenção ao parto, fundamentais para a redução da mortalidade neonatal precoce no Brasil ^8^ ^,^ ^15^ ^,^ ^21^.

Igualmente, a mortalidade materna permanece como uma pauta em evidência, e a sua redução é um desafio para a saúde pública global ^22^. O arrefecimento da mortalidade materna, inserida como meta nos Objetivos de Desenvolvimento do Milênio (ODM), não foi atingido por muitos países, incluindo o Brasil. A meta da Agenda 2030 foi adaptada à realidade nacional, redefinindo-a para reduzir a razão de mortalidade materna para 30 mortes por 100 mil nascidos vivos ^23^ ^,^ ^24^.

Apesar dos avanços na assistência reprodutiva e materno-infantil no país, a inadequação do pré-natal e a falta de integração da atenção primária com a assistência ao parto - ambos relacionados à mortalidade perinatal - ainda são problemas relevantes no país ^5^. Artigos anteriores evidenciaram importantes desigualdades na atenção pré-natal por subgrupos populacionais com base nos dados da primeira edição da *Pesquisa Nacional de Saúde* (PNS) ^25^ ^,^ ^26^ ^,^ ^27^. Nesse contexto, a informação de qualidade tem papel fundamental na análise da saúde materno-infantil, uma vez que contribui para o monitoramento da situação de saúde e oferece subsídios para planejamento, tomada de decisões baseadas em evidências e organização dos serviços de saúde ^28^.

Sendo assim, é necessário avaliar como as diretrizes estabelecidas pelo Ministério da Saúde referentes à assistência pré-natal estão sendo seguidas entre as gestantes brasileiras. O objetivo do presente estudo é analisar temporalmente indicadores relacionados ao pré-natal para estabelecer os avanços e os desafios a serem superados, com base nos dados das duas edições da PNS (2013 e 2019).

## Métodos

### Fonte de informações

A PNS é um estudo transversal, de âmbito nacional e base domiciliar, realizada pelo Ministério da Saúde em parceria com o Instituto Brasileiro de Geografia e Estatística (IBGE). Foi realizada nos anos de 2013 e 2019 e possibilita conhecer o perfil de saúde, as exposições e condições de risco da população brasileira, além de permitir a obtenção de indicadores para avaliação do desempenho do sistema de saúde. No que se refere às informações relacionadas à gestação, traz dados sobre a adequação do pré-natal, como: exames realizados durante o pré-natal, monitoramento clínico e obstétrico, orientações dadas às gestantes, peregrinação, local e assistência ao parto, entre outros. Neste estudo, foram utilizadas como fontes de informações as duas edições da PNS.

A amostra da PNS constitui uma subamostra da Amostra Mestra do Sistema Integrado de Pesquisas Domiciliares (SIPD) do IBGE e foi selecionada em três estágios ^29^. A PNS teve aprovação da Comissão Nacional de Ética em Pesquisa (CONEP) em julho de 2013 (protocolo nº 328.159) para a edição de 2013, e em agosto de 2019 (protocolo nº 3.529.376) para a edição de 2019.

A população-alvo do estudo é a de mulheres que tiveram algum parto nos últimos dois anos anteriores à pesquisa, ou seja, no período de 28 de julho de 2011 a 27 de julho de 2013 - para a PNS de 2013 -, e entre 28 de julho de 2017 e 27 julho de 2019 - para a PNS de 2019. Além disso, uma vez que na amostra da PNS 2013 a população com menos de 18 anos não foi incluída, no presente estudo, foram consideradas apenas as mulheres de 18 anos ou mais, para possibilitar a comparabilidade entre os anos.

Neste trabalho, foram utilizadas as informações do Módulo S - Atendimento Pré-natal, do questionário individual da PNS, referentes ao atendimento pré-natal no último parto.

### Variáveis

Para avaliar como as diretrizes propostas pelo Ministério da Saúde ^1^ relativas ao pré-natal estão sendo seguidas, foram consideradas as seguintes variáveis: realização do pré-natal (sim, não); trimestre de início do pré-natal (1º, 2º, 3º); número de consultas de pré-natal realizadas (1-3, 4-5, 6 ou mais); local de realização do pré-natal (público, privado, outro); profissional que atendeu no pré-natal (médico, enfermeiro, técnico/auxiliar de enfermagem); posse de caderneta/cartão da gestante (sim, não); tempo (em semanas) antes do parto em que foi realizada a última consulta de pré-natal (até 1, mais de 1 a 2, mais de 2, não sabe/não lembra); frequência de consultas em que foram feitas medidas de pressão arterial, do peso, da barriga, ausculta do coração do bebê e exame das mamas (todas, algumas, nenhuma); orientações sobre a maternidade de referência para o parto (sim, não, não sabe/não lembra); realização de exame de sangue durante o pré-natal (sim, não); realização de exame de urina durante o pré-natal (sim, não, não sabe/não lembra); realização do exame para sífilis durante o pré-natal (sim, não, não sabe/não lembra); recebimento do resultado do exame de sífilis antes do parto (sim, não recebeu o resultado/não foi informada antes do parto); resultado do teste de sífilis (positivo, negativo, recusou-se a responder); solicitação de exame para HIV/aids (sim, não, não sabe/não lembra); realização de exame para HIV/aids (sim, não); recebimento do resultado do exame para HIV/aids antes do parto (sim, não recebeu o resultado/não foi informada antes do parto); realização de exame para hepatite B (sim, não, não sabe/não lembra); recebimento do resultado do exame para hepatite B antes do parto (sim, não recebeu o resultado/não foi informada antes do parto); vacinação contra difteria e tétano (sim, não, não sabe/não lembra); número de doses difteria e tétano (1-2, 3 ou mais, não sabe/não lembra).

Algumas perguntas desse módulo não foram realizadas da mesma forma nas duas edições da pesquisa, no entanto, mantiveram o sentido das questões, e foi possível a comparação entre elas, conforme apresentado no Quadro 1. Além disso, algumas perguntas foram feitas somente na edição de 2019, o que não permitiu avaliar mudanças no período.

**Quadro 1:** Questões do Módulo S - Atendimento Pré-natal da *Pesquisa Nacional de Saúde*, 2013 e 2019.

2013	2019
Na última vez que a Sra. esteve grávida, a Sra. fez pré-natal?	Quando esteve grávida, fez alguma consulta de pré-natal?
Com quantas semanas de gravidez a Sra. iniciou o pré-natal?	Quantas tempo de gravidez tinha quando fez a primeira consulta pré-natal?
Quantas consultas de pré-natal a Sra. teve?	Quantas consultas de pré-natal fez durante essa gravidez?
Onde foi realizada a maioria das consultas do pré-natal?	Você fez a maioria das consultas do pré-natal em serviço de saúde de:
Quem a atendeu na maioria das consultas?	Nessa gravidez, quem a atendeu na maioria das consultas?
Na última vez que a Sra. esteve grávida a Sra. recebeu o cartão de pré-natal?	Nessa gravidez, você tinha uma caderneta/cartão da gestante?
Quanto tempo antes do parto a Sra. foi à última consulta do pré-natal?	Quanto tempo antes do parto a Sra. foi à última consulta do pré-natal?
Durante o pré-natal, em quantas consultas mediram sua pressão arterial?	Durante o pré-natal, em quantas consultas mediram sua pressão arterial?
Durante o pré-natal, em quantas consultas mediram o seu peso?	Durante o pré-natal, em quantas consultas mediram o seu peso?
Durante o pré-natal, em quantas consultas mediram a sua barriga?	Durante o pré-natal, em quantas consultas mediram sua barriga?
Durante o pré-natal, em quantas consultas ouviram o coração do bebê?	Durante o pré-natal, em quantas consultas ouviram o coração do bebê?
Durante o pré-natal, em quantas consultas examinaram suas mamas?	Durante o pré-natal, em quantas consultas examinaram suas mamas?
Durante o pré-natal a Sra. foi informada sobre a qual serviço de saúde a Sra. deveria ir no momento do parto?	Durante esse pré-natal você foi informada/orientada pelo serviço de saúde/profissional de saúde sobre a maternidade para qual deveria se dirigir no momento do parto?
Durante o pré-natal, a Sra. fez exame de sangue?	Nessa gravidez, você fez algum exame de sangue, sem considerar o teste de gravidez?
Durante o atendimento pré-natal a Sra. realizou exame de urina?	Nessa gravidez, você fez algum exame de urina, sem considerar o teste de gravidez?
Durante o atendimento pré-natal a Sra. realizou exame de sangue para sífilis?	Durante o pré-natal de (nome) foi realizado teste∕ exame para sífilis?
A Sra. recebeu o resultado do exame para sífilis antes do parto? (nas opções de resposta consta o resultado)	Recebeu ou foi informada sobre o resultado do teste/exame para sífilis antes do parto?
	Qual foi o resultado do teste/exame para sífilis?
Foi pedido exame de sífilis para o seu parceiro?	Foi solicitado teste/exame de sífilis para o seu parceiro(a)?
-	Durante o pré-natal foi realizado teste ou exame para hepatite B?
-	Recebeu o resultado da hepatite B antes do parto?
Durante seu pré-natal, foi solicitado o teste para HIV?	Durante o pré-natal foi solicitado o teste∕ exame para HIV/aids?
A Sra. fez o teste de HIV?	Nesse pré-natal foi realizado teste∕ exame para HIV/aids?
-	Recebeu o resultado do teste ou exame para HIV/aids antes do parto?
-	Quando esteve grávida tomou alguma injeção para prevenir o bebê contra difteria e tétano (mal dos sete dias) - dT ou dTpa?

### Análise dos dados

Foram construídos indicadores com as prevalências estimadas em cada uma das categorias das variáveis consideradas no estudo e respectivos intervalos de 95% de confiança (IC95%).

Os bancos de dados das duas edições da PNS foram relacionados verticalmente, adicionando-se os casos da edição de 2019 ao banco de dados de 2013. Posteriormente, uma nova variável de estratificação foi criada, considerando-se, conjuntamente, o ano de referência da pesquisa (2013 ou 2019) e a variável estrato original. A nova variável foi utilizada na definição do desenho amostral para estabelecer a estratificação das unidades primárias de amostragem, de modo a produzir estimativas corretas, tanto pontuais como de precisão.

Para comparar as distribuições proporcionais de cada variável nos anos de 2013 e 2019, foi aplicado o teste qui-quadrado de Pearson, ajustado pela correção de Rao-Scott, para levar em consideração o desenho complexo de amostragem da PNS. As prevalências e seus respectivos IC95% foram calculados no software SPSS versão 21 (https://www.ibm.com/), módulo *Complex Sample*, e o valor de p do teste qui-quadrado de Rao-Scott foi calculado no software Stata versão 14 (https://www.stata.com), módulo *Survey*.

## Resultados

Em 2013 foram analisadas 1.918 mulheres de 18 anos ou mais que tiveram parto nos últimos dois anos anteriores às edições da pesquisa, e em 2019, 2.910 mulheres.

Em 2013, 97,4% gestantes realizaram pelo menos uma consulta de pré-natal, e dessas, 88,5% iniciaram o pré-natal no 1º trimestre de gestação. Em 2019, os percentuais foram, respectivamente, de 98,1% e 88,8%, sem significância estatística. Quanto ao número de consultas, em 2013, 83,8% realizaram 6 ou mais consultas, e em 2019, 87,3%, com aumento estatisticamente significativo (p = 0,0340) (Tabela 1).

**Tabela 1:** Proporção de gestantes segundo procedimentos realizados no pré-natal. *Pesquisa Nacional de Saúde* (PNS), 2013 e 2019

Variável	PNS 2013			PNS 2019			Valor de p *
	n	%	IC95%	n	%	IC95%	
Realização de consulta de pré-natal no último parto							
Sim	1.571	97,4	96,3-98,1	2.819	98,1	97,3-98,6	0,1851
Não	43	2,6	1,9-3,7	55	1,9	1,4-2,7	
Trimestre de início do pré-natal							
1º	1.325	88,5	86,0-90,5	2.503	88,8	87,0-90,3	0,8491
2º	161	10,7	8,8-13,0	287	10,2	8,7-11,9	
3º ou mais	13	0,8	0,4-1,9	29	1,0	0,6-1,6	
Consultas de pré-natal realizadas							
1-3	57	3,6	2,8-4,7	98	3,6	2,6-4,9	0,0340
4-5	199	12,6	10,5-15,2	251	9,1	7,5-11,1	
6 ou mais	1.315	83,8	81,1-86,1	2.408	87,3	85,1-89,2	
Local de realização do pré-natal							
Público	1.129	71,8	68,9-74,6	1.974	70,0	67,0-72,8	0,4077
Privado	431	27,4	24,7-30,3	833	29,6	26,7-32,5	
Outro	12	0,8	0,5-1,2	12	0,4	0,1-1,4	
Profissional que atendeu na maioria das consultas de pré-natal							
Médico(a)	1.123	71,5	68,8-73,9	2.046	72,6	70,0-75,0	0,1250
Enfermeiro(a)	431	27,4	25,0-30,0	754	26,7	24,3-29,3	
Técnico(a)/Auxiliar de enfermagem	13	0,8	0,5-1,4	19	0,7	0,4-1,2	
Outro	4	0,3	0,1-0,8	0	0,0	-	
Posse de caderneta/cartão da gestante							
Sim	1.501	95,5	94,2-96,6	2.717	96,4	94,9-97,4	0,3550
Não	70	4,5	3,4-5,8	103	3,6	2,6-5,1	
Quanto tempo antes do parto ocorreu a última consulta de pré-natal							
Até 1 semana	759	48,3	45,0-51,6	1.737	61,6	58,7-64,4	0,0000
Mais de 1 semana a 2 semanas	431	27,4	24,7-30,4	568	20,1	17,7-22,8	
Mais de 2 semanas	312	19,9	17,7-22,3	401	14,2	12,5-16,2	
Não sabe/Não lembra	69	4,4	3,3-5,9	114	4,1	3,0-5,5	

IC95%: intervalo de 95% de confiança.

* Nível descritivo do teste qui-quadrado de Pearson, ajustado pela correção de Rao-Scott, de comparação das distribuições proporcionais de cada variável nos anos de 2013 e 2019.

Entre 2013 e 2019, a proporção de mães que realizaram pré-natal em setor privado variou de 27,4% para 29,6%. E o percentual de realização de consultas de pré-natal atendidas por médico, de 71,5% (2013) para 72,6% (2019), não havendo aumento significativo (Tabela 1). Igualmente, para a posse de caderneta/cartão da gestante, não houve diferença estatisticamente significativa (p = 0,3550).

Em 2013, 48,3% das mães realizaram a última consulta do pré-natal em até uma semana antes do parto, enquanto em 2019, a proporção foi de 61,6%, representando um aumento significativo (p < 0,001) (Tabela 1).

Em relação aos exames clínico-obstétricos realizados durante o pré-natal, comparando os resultados entre 2013 e 2019, foi possível observar que a medida da pressão arterial (91,9%; 94,7%), a medida de peso corporal (91,9%; 95,3%), a medida da barriga da gestante (79,2%; 85,6%) e a ausculta cardíaca do bebê (79,4%; 81,5%) realizadas em todas as consultas de pré-natal, apresentaram aumento significativo no período. Quanto à realização de exame nas mamas, em nenhuma das consultas, o percentual aumentou significativamente de 37,1% para 64,6% (p = 0,0000) (Tabela 2). Já a prevalência de mulheres que receberam orientação sobre o serviço de saúde de referência para a realização do parto aumentou significativamente de 75,1%, em 2013, para 83%, em 2019 (p = 0,0000) (Tabela 2).

**Tabela 2:** Proporção de gestantes segundo realização de exames clínico-obstétricos e orientações recebidas durante o pré-natal. *Pesquisa Nacional de Saúde* (PNS), 2013 e 2019.

Variável	PNS 2013			PNS 2019			Valor de p *
	n	%	IC95%	n	%	IC95%	
Em quantas consultas mediram a pressão arterial?							
Todas	1.444	91,9	90,0-93,5	2.669	94,7	93,4-95,7	0,0002
Algumas	125	7,9	6,4-9,8	131	4,7	3,7-5,9	
Nenhuma	3	0,2	0,1-0,4	19	0,6	0,4-1,1	
Em quantas consultas mediram o peso?							
Todas	1.445	91,9	90,1-93,5	2.686	95,3	93,9-96,3	0,0022
Algumas	122	7,8	6,2-9,7	121	4,3	3,3-5,5	
Nenhuma	5	0,3	0,1-0,8	13	0,4	0,2-1,4	
Em quantas consultas mediram a barriga?							
Todas	1.245	79,2	76,8-81,4	2.412	85,6	83,5-87,4	0,0004
Algumas	253	16,1	13,9-18,6	324	11,5	9,8-13,4	
Nenhuma	73	4,7	3,3-6,6	83	2,9	2,4-3,7	
Em quantas consultas ouviram o coração do bebê?							
Todas	1.248	79,4	76,8-81,8	2.297	81,5	79,4-83,3	0,0114
Algumas	283	18,0	15,4-20,8	494	17,5	15,7-19,5	
Nenhuma	41	2,6	1,6-4,2	29	1,0	0,7-1,5	
Em quantas consultas examinaram as mamas?							
Todas	593	37,7	34,6-41,0	617	21,9	19,6-24,4	0,0000
Algumas	396	25,2	22,1-28,5	381	13,5	11,3-16,1	
Nenhuma	583	37,1	34,1-40,2	1.821	64,6	61,5-67,6	
Foi orientada sobre a maternidade para qual deveria se dirigir no momento do parto?							
Sim	1.181	75,1	72,4-77,7	2.341	83,0	81,0-84,9	0,0000
Não	391	24,9	22,3-27,6	448	15,9	14,1-17,9	
Não sabe/Não lembra	0	0,0	-	30	1,1	0,6-1,8	

IC95%: intervalo de 95% de confiança.

* Nível descritivo do teste qui-quadrado de Pearson, ajustado pela correção de Rao-Scott, de comparação das distribuições proporcionais de cada variável nos anos de 2013 e 2019.

Sobre a realização de exames durante o pré-natal (Tabela 3), entre 2013 e 2019, houve decréscimos significativos entre as gestantes que realizaram exame de sangue, de 97,3% para 94,3% (p = 0,0003), e exame de urina, de 98,1% para 93,3% (p = 0,0000). Quanto à realização de teste de sífilis e de HIV durante o pré-natal, houve aumentos significativos (p = 0,0000), de 66,4% para 79,7% e de 95,5% para 99,3%, respectivamente. Não houve diferença significativa na proporção de mulheres que não receberam ou não foram informadas sobre o resultado do exame de sífilis antes do parto. A proporção de gestantes com exames positivos para sífilis foi de 1,2%, em 2013, e de 0,9%, em 2019, sem variação significativa no período. O percentual de solicitação do teste de HIV durante o pré-natal também não apresentou diferença significativa entre 2013 e 2019. O recebimento do resultado do teste de HIV antes do parto só foi questionado na edição de 2019 da PNS e apresentou um percentual de 97,7% (Tabela 3).

**Tabela 3:** Proporção de mulheres segundo realização de exames de infecções sexualmente transmissíveis durante o pré-natal. *Pesquisa Nacional de Saúde* (PNS), 2013 e 2019.

Variáveis	PNS 2013			PNS 2019			Valor de p *
	n	%	IC95%	n	%	IC95%	
Realização de exame de sangue durante o pré-natal							
Sim	1.529	97,3	96,2-98,1	2.660	94,3	93,1-95,4	0,0003
Não	42	2,7	1,9-3,8	159	5,7	4,6-6,9	
Realização de exame de urina durante o pré-natal							
Sim	1.541	98,1	97,3-98,6	2.630	93,3	91,8-94,5	0,0000
Não	30	1,9	1,4-2,7	183	6,5	5,3-8,0	
Não sabe/Não lembra	0	0,0	-	6	0,2	0,1-0,7	
Realização de teste ou exame para sífilis durante o pré-natal							
Sim	1.016	66,4	63,0-69,6	2.247	79,7	77,5-81,7	0,0000
Não	293	19,2	16,9-21,6	324	11,5	9,9-13,4	
Não sabe/Não lembra	220	14,4	11,7-17,6	249	8,8	7,5-10,3	
Recebeu ou foi informada sobre o resultado do teste ou exame para sífilis antes do parto							
Sim	997	98,1	97,1-98,8	2.174	96,8	95,4-97,7	0,0518
Não recebeu o resultado/Não foi informada antes do parto	19	1,9	1,2-2,9	72	3,2	2,3-4,6	
Resultado do teste ou exame para sífilis							
Positivo	12	1,2	0,5-2,8	21	0,9	0,5-1,8	0,6052
Negativo	985	98,8	97,2-99,5	2.145	98,7	97,7-99,2	
Recusou-se a responder	0	-	-	9	0,4	0,1-1,1	
Foi solicitado o teste ou exame para HIV/aids durante o pré-natal							
Sim	1.400	89,1	87,0-90,9	2.535	89,9	88,3-91,4	0,4203
Não	100	6,4	5,2-7,8	186	6,6	5,5-7,9	
Não sabe/Não lembra	71	4,5	3,2-6,4	98	3,5	2,5-4,7	
Realização do teste ou exame para HIV/aids durante o pré-natal							
Sim	1.337	95,5	93,4-96,9	2.518	99,3	98,9-99,6	0,0000
Não	63	4,5	3,1-6,6	17	0,7	0,4-1,1	
Recebeu o resultado do teste ou exame para HIV/aids antes do parto **							
Sim				2.461	97,7	96,5-98,5	-
Não recebeu o resultado/Não foi informada antes do parto				58	2,3	1,5-3,5	
Realização de teste ou exame para hepatite B durante o pré-natal **							
Sim				2.374	84,2	82,1-86,1	-
Não				242	8,6	7,0-10,4	
Não sabe/Não lembra				203	7,2	6,1-8,5	
Recebeu ou foi informada sobre o resultado do teste ou exame para hepatite B antes do parto **							
Sim				2.289	96,4	94,8-97,5	-
Não recebeu o resultado/Não foi informada antes do parto				86	3,6	2,5-5,2	

IC95%: intervalo de 95% de confiança.

* Nível descritivo do teste qui-quadrado de Pearson, ajustado pela correção de Rao-Scott, de comparação das distribuições proporcionais de cada variável nos anos de 2013 e 2019;

** Pergunta realizada somente em 2019.

Algumas questões como a realização de exame para hepatite B e vacinação contra difteria e tétano só foram incluídas na edição de 2019 da PNS. Nota-se que 84,2% das mulheres realizaram exame para hepatite B durante o pré-natal, e dessas, 96,4% receberam o resultado antes do parto (Tabela 3). Com relação à vacinação contra difteria e tétano, 63,2% das mulheres receberam pelo menos uma dose, entretanto 11,7% não sabem ou não lembram se receberam a vacina. Entre as que declararam ter recebido a vacina, 57,4% receberam 1-2 doses, e 11,8%, 3 ou mais (Tabela 4).

**Tabela 4:** Proporção de mulheres segundo vacinação contra difteria e tétano durante o pré-natal. *Pesquisa Nacional de Saúde* (PNS) de 2019.

Variável	PNS 2019		
	n	%	IC95%
Vacinação contra difteria e tétano (mal dos sete dias) - dT ou dTpa			
Sim	1.780	63,2	60,2-66,0
Não	707	25,1	22,5-27,9
Não sabe/Não lembra	332	11,7	9,9-14,0
Doses da vacina contra difteria e tétano (mal dos sete dias) - dT ou dTpa entre as mulheres que receberam a vacina			
1-2	1.022	57,4	53,8-60,8
3 ou mais	211	11,8	9,8-14,3
Não sabe/Não lembra	548	30,8	27,7-34,0

IC95%: intervalo de 95% de confiança.

## Discussão

Este estudo analisou fatores relacionados à qualidade da assistência ao pré-natal, com base nas duas edições da PNS, a partir das informações de mulheres participantes na pesquisa que tiveram filho nos últimos dois anos anteriores à pesquisa. Com vistas a estabelecer os avanços e os persistentes problemas no atendimento pré-natal, o artigo foi focado em analisar a tendência temporal de realização dos procedimentos clínico-obstétricos preconizados pelo Ministério da Saúde e das orientações dadas às gestantes na atenção pré-natal.

Os achados do presente estudo mostraram uma tendência de melhora do pré-natal entre 2013 e 2019, com destaque para o aumento do número de consultas de pré-natal. Em estudo utilizado - dados da pesquisa *Nascer no Brasil* ^30^ - nos anos de 2011 e 2012, foram encontrados resultados semelhantes, como boas coberturas de pré-natal (98%), nas quais 73,1% das mulheres compareceram a seis ou mais consultas. Análise de inquéritos de saúde realizados em 1986, 1996, 2006 e 2013, no Brasil, mostrou aumentos importantes na proporção de gestantes que realizaram quatro ou mais consultas de pré-natal, indicando avanços progressivos na cobertura do pré-natal após a implementação do Sistema Único de Saúde (SUS) ^27^. Apesar dos aumentos encontrados, a Organização Mundial da Saúde (OMS), desde 2016, recomenda um mínimo de oito consultas, e a Rede Cegonha, desde 2011, recomenda sete ou mais consultas, indicando que é necessário atualizar o protocolo do Ministério da Saúde ^31^ ^,^ ^32^.

Quanto ao mês de início do pré-natal, um estudo nos Estados Unidos ^33^ mostrou que a busca precoce de atenção pré-natal está associada a desfechos positivos para mulheres e bebês, e que atrasos no reconhecimento da gravidez e na realização da primeira consulta pré-natal representam oportunidades perdidas de iniciar o pré-natal precocemente. O estudo apontou desigualdades sociodemográficas e barreiras de acesso aos serviços de saúde como fatores para cuidados tardios. No Brasil, diferentemente dos Estados Unidos, é preconizado o início do pré-natal no primeiro trimestre, e não houve aumento significativo no período de 2013 a 2019.

De acordo com o Ministério da Saúde, deve ser assegurado um acompanhamento periódico e contínuo de todas as gestantes durante toda a gestação ^3^. As consultas devem seguir intervalos preestabelecidos de acordo com o tempo de gestação (até a 12ª semana gestacional, deve ocorrer a primeira consulta; até a 28ª semana, as consultas devem ser mensais; da 28ª até a 36ª semana, devem ser quinzenais; e da 36ª a 41ª, semanais) ^3^. Neste sentido, cabe destacar o avanço observado com os dados da PNS, que mostraram melhoria importante com cerca de 60% das mulheres realizando a última consulta até uma semana antes do parto, em 2019.

Com relação à posse do caderneta/cartão da gestante, não foi observada diferença significativa no período analisado. O Ministério da Saúde recomenda seu uso desde 1988 como instrumento essencial para registrar os cuidados mínimos no pré-natal e favorecer a integração entre os diversos níveis da assistência. Contudo, o fornecimento da caderneta não abrange a totalidade das gestantes, e a qualidade no preenchimento das informações não é boa em alguns locais do país ^34^.

Quanto aos exames clínicos-obstétricos, as medições de pressão arterial, peso, barriga e ausculta cardíaca do bebê, avaliadas neste estudo, apresentaram aumentos significativos ente 2013 e 2019. No entanto, alguns indicadores apresentaram tendência de piora, como a realização de exames de sangue, urina e de mamas, e este, com o aumento para a não realização em nenhuma das consultas em 2019. Um estudo anterior que analisou indicadores da qualidade da assistência ao pré-natal em uma coorte de nascimento de Pelotas (Rio Grande do Sul) em 2015 apresentou padrões semelhantes aos encontrados na PNS. Foram identificadas elevadas proporções nos exames de medição de pressão arterial e peso e, por outro lado, menores proporções nos exames de mamas e elevados índices de início tardio do pré-natal (no terceiro trimestre da gestação) ^35^.

No âmbito das ações da atenção pré-natal, a unidade de atenção primária deve garantir o pré-natal de forma continuada, incluindo prevenção, diagnóstico, tratamento de doenças e suporte social, cultural e psicológico à gestante ^11^. Assim, durante o pré-natal, o exame clínico das mamas é realizado para detectar anormalidades e possíveis lesões malignas palpáveis em um estágio precoce de evolução, alertar para os sintomas e fatores de risco e promover o aleitamento materno ^3^. Contudo, com o tempo, alguns procedimentos podem estar sendo negligenciados pelos profissionais de saúde com maior frequência, como o exame clínico de mamas durante o pré-natal ^36^.

Quanto aos exames relativos às infecções sexualmente transmissíveis, a PNS apresentou tendência de aumento na realização dos exames de sífilis e HIV, durante o pré-natal, indicando a possibilidade de diagnóstico precoce e a garantia na oportunidade do tratamento em tempo adequado, corroborando achados de outros estudos ^37^ ^,^ ^38^. Entretanto, esse aumento no diagnóstico não resultou na redução dos casos de sífilis congênita, o que indica a necessidade de revisão dos processos de abastecimento de penicilina benzatina na atenção primária à saúde ^39^ ^,^ ^40^. Problemas como a falta de tratamento adequado, o diagnóstico tardio no pré-natal e a demora na obtenção do resultado do exame foram determinantes para o aumento dos casos de sífilis congênita no Brasil ^41^. Segundo dados do *Boletim Epidemiológico - Sífilis* ^42^, em 2022, 5,7% de casos de sífilis em gestantes não receberam tratamento, variando de 4,2% na Região Sudeste a 8,3% na Região Nordeste.

Um estudo que utilizou dados da avaliação externa do Programa Nacional de Melhoria do Acesso e da Qualidade da Atenção Primária (PMAQ) - que descreve uma série de indicadores de qualidade do pré-natal -, indicou semelhança com alguns resultados do presente artigo, como a evolução positiva para os exames de HIV e sífilis ^43^. O aumento foi associado à maior disponibilidade de testes rápidos nas unidades de saúde e à melhoria na estrutura de recursos humanos. Em 2012, a Rede Cegonha instituiu a realização de testes rápidos para a detecção de HIV e sífilis na atenção básica, sendo essa uma provável explicação para o aumento dos testes rápidos na rede de atenção à saúde ^41^ ^,^ ^44^. Conforme o *Boletim Epidemiológico - Sífilis* ^42^, de 2023, o percentual de gestantes diagnosticadas com sífilis no primeiro trimestre aumentou de 23,2% em 2012 para 46,1% em 2022.

Em relação à proporção de gestantes que tiveram orientação sobre o local do parto, apesar do aumento significativo entre 2013 e 2019, uma parcela considerável de gestantes ainda não recebe esta orientação, o que pode resultar na peregrinação pela procura dos serviços de saúde no momento do parto. O atraso na chegada da parturiente ao serviço de saúde é um dos principais fatores associados a desfechos desfavoráveis para a gestante e para o feto, com maior ocorrência de *near miss* materno e óbitos neonatais ^21^ ^,^ ^45^ e permanecendo como desafio para redução da mortalidade infantil, particularmente a perinatal ^5^.

No Brasil, a organização da rede de atenção à gestante, com fluxo estabelecido de referência e contrarreferência dos serviços, evitando a peregrinação dessas mulheres, e a realização de um parto humanizado e seguro tem contribuído para evitar desfechos negativos graves ^6^. No entanto, essa ampliação desencadeou novos desafios ao sistema de saúde, como a dificuldade para estabelecer fluxos integrados e contínuos de comunicação, além do aumento da demanda por atendimentos e procedimentos em diferentes níveis de complexidade ^46^.

Os indicadores relacionados à vacinação merecem também atenção. Os resultados da PNS mostraram que 63% se vacinaram contra difteria e tétano na gestação, mas somente 12% das gestantes realizou o esquema vacinal completo (três doses). No entanto, cabe ressaltar o viés de memória por parte das entrevistadas, uma vez que há gestantes que não souberam responder se foram vacinadas e entre aquelas que foram, não souberam responder sobre o número de doses feitas. Com exceção a esses casos, pode-se entender que as demais gestantes realizaram pelo menos a dose de reforço. No caso da PNS, não houve possibilidade de inferir a tendência temporal da vacinação, uma vez que esse tópico foi perguntado apenas em 2019, mas no estudo de Tomasi et al. ^43^, observou-se uma redução nas proporções de mulheres que receberam a vacina contra tétano. Entre os ciclos do estudo, a cobertura da vacinação contra tétano teve uma redução anual de 1,2%.

### Limitações

A PNS, com o suporte de um entrevistador capacitado para criar adequada abordagem e comunicação aos entrevistados, tem a vantagem de perguntar sobre os procedimentos feitos durante o pré-natal de maneira detalhada. Contudo, apresenta limitações por ser baseada em respostas referidas durante a entrevista domiciliar, podendo estar atrelado ao viés de memória da entrevistada, à superestimação dos procedimentos realizados diante do que é esperado, ou à subestimação pela falta de compreensão e interpretação das questões durante a entrevista - ou até pelo constrangimento em responder a algumas questões. Destaca-se que a análise de registros (caderneta vacinal) poderia auxiliar em uma avaliação mais fidedigna da vacinação das mulheres gestantes.

Adicionalmente, a diferença na elaboração de algumas perguntas realizadas em 2013 e em 2019, embora sobre mesmo conteúdo, tiveram formas de captação distintas, impossibilitando algumas análises comparativas.

Outra questão refere-se aos itens de resposta “*nenhuma, algumas, todas*”, que aparecem em perguntas como “Em quantas consultas foram realizadas medidas de pressão arterial?”. Esses itens de respostas são limitantes, pois não descrevem especificamente o número de consultas, restringindo possíveis análises. Desse modo, sugerimos, para a próxima edição da PNS, uma adequação desses itens para melhor análise das informações da pesquisa de acordo com os protocolos vigentes do pré-natal pelo Ministério da Saúde.

Além disso, outras práticas recomendadas no pré-natal, suplementação com sulfato ferroso e ácido fólico, e orientações como aleitamento materno, preparação para o parto, e cessação do tabagismo e uso de álcool não constam do questionário da PNS e não puderam ser analisadas.

## Conclusões

A análise da assistência ao pré-natal, fundamentada nos dados das duas edições da PNS, revela progressos na cobertura e na qualidade do atendimento às gestantes no Brasil, assim como na adesão às diretrizes fundamentais do SUS.

Entre os avanços significativos, destaca-se o aumento do número de consultas e da realização de exames clínicos e laboratoriais, como os testes para sífilis e HIV. Esses progressos são essenciais para garantir um acompanhamento adequado e de qualidade às gestantes. Contudo, houve um decréscimo na realização de alguns exames, como os de sangue, urina e mamas. Adicionalmente, não houve diferença significativa na proporção de gestantes que iniciaram o pré-natal no primeiro trimestre, indicando que alguns aspectos do cuidado pré-natal ainda necessitam de maior atenção.

Persistem desafios no que diz respeito à integração entre a atenção primária e a assistência ao parto, como a falta de orientação sobre o local de parto, afetando negativamente a mortalidade materna e infantil. A organização de uma rede assistencial integrada desde a atenção primária até os serviços de alta complexidade é essencial para assegurar a integralidade da atenção à criança e à gestante e deve se basear nos princípios garantidos na *Constituição Federal*, no *Estatuto da Criança e do Adolescente* (ECA) e no SUS, como o direito de acesso aos serviços de saúde, hierarquizados, com enfoque da integralidade do indivíduo, garantindo a resolubilidade adequada e promovendo a equidade.

Os achados do presente estudo ressaltam a importância de fortalecer as diretrizes do Ministério da Saúde, garantindo que todas as gestantes tenham acesso a um acompanhamento adequado desde o início da gestação. A melhoria contínua do pré-natal é essencial para a redução da mortalidade infantil, fetal e materna e para promover um desenvolvimento saudável das crianças e uma maternidade segura. Portanto, é crucial o incentivo e o reforço de políticas que ampliem o acesso ao pré-natal de qualidade, integrando serviços de saúde e promovendo a educação das gestantes sobre os cuidados necessários durante a gravidez. Assim, asseguramos o direito à saúde e à vida, contribuindo para o alcance das metas estabelecidas nos ODS.
